# Increased somatic mosaicism in autosomal and X chromosomes for suicide death

**DOI:** 10.1038/s41380-024-02718-y

**Published:** 2024-08-30

**Authors:** Ikuo Otsuka, Shunsuke Uchiyama, Toshiyuki Shirai, Xiaoxi Liu, Motonori Takahashi, Yoichiro Kamatani, Chikashi Terao, Akitoyo Hishimoto

**Affiliations:** 1 Department of Psychiatry, Kobe University Graduate School of Medicine, Kobe, Japan; 2 Laboratory for Statistical and Translational Genetics, RIKEN Center for Integrative Medical Sciences, Yokohama, Japan; 3 Department of Allergy and Rheumatology, Nippon Medical School, Tokyo, Japan; 4 Clinical Research Center, Shizuoka General Hospital, Shizuoka, Japan; 5 Division of Legal Medicine, Department of Community Medicine and Social Health Science, Kobe University Graduate School of Medicine, Kobe, Japan; 6 Laboratory of Complex Trait Genomics, Graduate School of Frontier Sciences, The University of Tokyo, Tokyo, Japan; 7 The Department of Applied Genetics, The School of Pharmaceutical Sciences, University of Shizuoka, Shizuoka, Japan

**Keywords:** Genetics, Psychology

## Abstract

Mosaic chromosomal alterations (mCAs) are classified as mosaic deletions (loss), copy-neutral loss of heterozygosity (CN-LOH), and duplications (gain), attracting special attention as biological aging-related acquired genetic alterations. While these mCAs have been linked with aging and various diseases, no study has investigated their association with suicide risk which is associated with abnormal biological aging. Here, we examined the association between suicide deaths and mCAs, including mosaic loss of the X (mLOX) and Y chromosomes, by leveraging blood-derived single nucleotide polymorphism-array data. The first (410 suicide decedents and 88,870 controls) and the second (363 suicide decedents and 88,870 controls) cohorts were analyzed and integrated using meta-analyses (773 suicide decedents and 177,740 controls). Total mCAs in autosomal chromosomes were significantly increased in suicide (p = 1.28 × 10^−6^, odds ratio [OR] = 1.78), mostly driven by loss (p = 4.05 × 10^−9^, OR = 2.70) and gain (p = 1.08 × 10^−3^, OR = 2.23). mLOX were significantly increased in female suicide (p = 2.66 × 10^−21^, OR = 4.00). The directions of effects of all mCAs in autosomal and sex chromosomes on suicide were the same in the first and second sets. Subgroup analyses suggest that our findings were mostly driven by suicide itself, and not confounded by comorbid psychiatric disorders or physical diseases, smoking status, sample location, or postmortem sample status. In conclusion, we provide the first evidence for aberrant mCAs in somatic autosomal and X chromosomes in suicide, which may contribute to an improved understanding of the genomic pathophysiology underlying suicide.

## Introduction

Suicide is a prevalent global public health issue, resulting in an estimated one million deaths annually. There is evidence for high heritability in suicidal behavior, with an estimated h^2^ of 30–55% based on twin, family, and adoption studies in European populations [1–3]. Recent large-scale genome-wide association studies (GWASs), including ours on the East Asian population, have yielded promising and replicable results [4–7]. Furthermore, there is now increasing evidence that aberrant biological aging, including accelerated epigenetic aging and telomere shortening, are also significantly associated with the risk of suicide [8–12].

Lifetime somatic mutations have attracted special attention as acquired genetic alterations. The genome of somatic cells mutates at a rate twice that of the germline in humans [13, 14]. Therefore, the genome of somatic cells differs from other cells, even within the same tissue. This phenomenon is termed “mosaic chromosomal alterations” (mCAs). mCAs are classified as mosaic deletions (loss), copy-neutral loss of heterozygosity (CN-LOH), and duplications (gain) [15]. These phenomena occur in the autosomal chromosomes as well as in the sex chromosomes [16–21]. The occurrence of these mCAs is often more frequent in older individuals, potentially raising the risk of multiple diseases, including haematological malignancies and overall mortality [22–24]. Recently, Loh et al. and our group developed a new method to detect each type of mCA (loss, CN-LOH, and gain) using blood-derived single nucleotide polymorphism (SNP)-array data both in the European-ancestry and East Asian-ancestry populations, respectively [15, 23]. Given this highly sensitive algorithm that leverages haplotype information based on SNP-array to detect mCAs, somatic mosaicism has been the focus of several recent efforts that took advantage of key technological innovations to elucidate aging and disease directly in humans, not only haematological traits but also other non-hematological traits including psychiatric disorders such as autism and schizophrenia [25–27]. Furthermore, somatic mosaicism in the brain is proposed to contribute to neuronal diversity [28, 29]. However, no study has investigated whether aberrant mCAs are involved in the risk of suicide. Here, we aimed to investigate the association between suicide deaths and mCAs by leveraging blood-derived SNP array data from the largest suicidal and non-suicidal samples in the East Asian population.

## Subjects and Methods

### Study cohort

This study was conducted in accordance with the principles of the Declaration of Helsinki. The main study cohort comprised two datasets. DNA was extracted from blood samples for all individuals. For suicide cases, we collected the postmortem blood samples from 434 individuals who died by suicide between June 1996 and July 2012 (for the first set) and another 405 individuals who died by suicide between August 2012 and February 2017 (for the second set). Then, we genotyped them using Illumina HumanOmniExpress BeadChips for the first set and HumanOmniExpressExome BeadChips for the second set. Written informed consent was obtained from the families of all suicide decedents for postmortem blood analysis, and approval was obtained from the Ethics Committee for Genetic Studies of Kobe University Graduate School of Medicine. Autopsies of suicide decedents were conducted at the Division of Legal Medicine in the Department of Community Medicine and Social Health Science at Kobe University Graduate School of Medicine. The verdict of “suicide death” was made through discussion with the Medical Examiner’s Office of the Hyogo Prefecture and the Division of Legal Medicine in Kobe University Graduate School of Medicine. To obtain background information on suicide decedents, psychological autopsies based on their medical records and bereaved family interviews were conducted, where available [6]. For controls, we used about 200,000 Biobank Japan (BBJ) samples. BBJ is a large registry of patients diagnosed with 47 common diseases. These 47 diseases did not include psychiatric disorders such as mood disorders. They collected clinical information, blood DNAs, and serum samples from 66 hospitals between 2003 and 2007. Genotyping of BBJ samples was performed using Illumina Human OmniExpress v1.0, Human OmniExpressExome v1.0 or Human OmniExpressExome v1.2. All of the study participants were diagnosed by medical doctors as described elsewhere [15, 30]. We complied with all relevant ethical regulations. This project was approved by the Ethics Committee of the RIKEN Center for Integrative Medical Sciences and Institute of Medical Sciences, University of Tokyo. Written informed consent was obtained from all participants.

In addition to the above-mentioned samples of the main cohort, the DNA samples from the blood of 138 healthy living participants and from the postmortem blood of 91 non-suicide decedents were collected at Kobe University Graduate School of Medicine and used as a supplementary cohort. These were genotyped using Infinium OmniExpress24 v1.4 to test potential confounding factors, such as sample location where blood sampling and DNA extraction were performed and biological status of blood tissues (from living participants or postmortem). Written informed consent was obtained from the healthy living participants and families of the decedents, and approval was obtained from the Ethics Committee for Genetic Studies of Kobe University Graduate School of Medicine.

### Calling mosaic events

To detect mosaic chromosomal alterations, we used the MoChA WDL pipeline　(https://github.com/freeseeek/mochawdl). MoChA is a free software to detect mosaic events from DNA microarray data. Illumina raw IDAT files of all samples were used as the input files. The MoChA pipeline first used Illumina’s Gencall algorithm to normalize and call genotypes from raw intensities and then converted llumina genotyping data to VCF files using the bcftools gtc2vcf plugin. The genotyping phasing was performed using SHAPEIT4 [31] with all samples together. Finally, we could detect mosaic chromosomal alterations using MoChA. We removed the genetically identical samples (PI_HAT > 0.9 estimated by PLINK1.9) [32] and the samples that were principal component analysis outliers from East Asian clusters in a plot in which we mapped the BBJ samples with 1KG populations (Fig. S1). We also removed the samples with genotyping call rate < 0.97 or the baf_auto > 0.03, which is indicative of poor DNA quality or contamination, and samples with genotype-phenotype sex discordance. To improve the phasing and imputation accuracy, we removed the variants with call rate < 0.97, those falling in segmental duplications with low divergence (<2%), those with excess heterozygosity (p < 1.0 × 10^−6^, Hardy-Weinberg equilibrium test), and those with allele frequency differences of > 3.0% from those in the imputation panel. After the mosaic calling, we removed the events satisfying any of the following criteria: (1) germline copy number polymorphism (CNPs) (2) constitutional mosaic events (lod_baf_phase <10), or (3) germline duplications (length < 500 kbp and relative coverage > 2.5). To filter out XXY and XXX samples, we restricted mLOX and mLOY with estimated ploidy less than 2.5. Finally, we obtained information of various somatic mosaicism [all autosomal mCAs (a total of loss, CN-LOH, and gain), loss, CN-LOH and gain in autosomal chromosomes, and mLOX and mLOY in sex chromosomes] for the samples remaining after filtering. We confirmed the presence or absence of mCAs in each individual. The 2022-12-14 version of MoChA was used to detect mosaic events.

For the main cohort, the first cohort consisted of 410 suicide decedents (275 males and 135 females) and 88,870 controls (47,647 males and 41,223 females), and the second cohort consisted of 363 suicide decedents (240 males, 123 females) and 88,870 controls (47,646 males, 41,224 females). For the first cohort, we used the Illumina Human OmniExpress v1.0 array for 410 suicide decedents and 15,938 controls, Human OmniExpressExome v1.0 array for 17,262 controls, and the Human OmniExpressExome v1.2 array for 55,670 controls. As for the used array of the second cohort, Human OmniExpress v1.0 array for 16,193 controls, Human OmniExpressExome v1.0 array for 17,158 controls, and Human OmniExpressExome v1.2 array for 363 suicide decedents and 55,519 controls. We randomly assigned the BBJ samples to two cohorts, matching age and sex, to keep as close to the number of participants as possible. In addition to the above-mentioned main two cohorts, a supplementary cohort consisting of 138 healthy participants (77 males and 61 females) and 91 non-suicide decedents (53 males and 38 females) were also used to test potential sample location and postmortem tissue biases. We removed the samples with a past medical history of haematological malignancy for the controls. The demographic data for the main two cohorts are listed in Table 1 and those for the supplementary cohort are listed in Supplementary Table S1. An overview of the whole study cohort is shown in Fig. S2.

**Table 1:** Demographic data of suicide decedents and controls in 1st and 2nd set.

	Suicide decedents	Controls
1st set		
Number of subjects (M/F)	410 (275/135)	88,870 (47,647/41,223)
Age (mean ± standard deviation, years)	M: 50.49 ± 16.91 F: 49.55 ± 18.72	M: 63.28 ± 13.38 F: 62.48 ± 15.00
Array		
Illumina Human OmniExpress v1.0	410	15,938
Illumina Human OmniExpressExome v1.0	0	17,262
Illumina Human OmniExpressExome v1.2	0	55,670
Comorbid psychiatric disorder		
Psychotic disorders	27 (22/5)	
Mood disorders	158 (80/78)	
Substance-related disorders	14 (12/2)	
Personality disorders	5 (1/4)	
Anxiety disorders	10(4/6)	
Other psychiatric diagnosis	11(8/3)	
No psychiatric diagnosis	145 (117/28)	
Unknown diagnosis	40 (31/9)	
2nd set		
Number of subjects (M/F)	363 (240/123)	88,870 (47,646/41,224)
Age (mean ± standard deviation, years)	M: 53.42 ± 18.40 F: 55.59 ± 16.96	M: 63.29 ± 13.40 F: 62.48 ± 14.99
Array		
Illumina Human OmniExpress v1.0	0	16,193
Illumina Human OmniExpressExome v1.0	0	17,158
Illumina Human OmniExpressExome v1.2	363	55,519
Comorbid psychiatric disorder		
Psychotic disorders	23 (17/6)	
Mood disorders	138 (67/71)	
Substance-related disorders	11 (10/1)	
Personality disorders	3 (0/3)	
Anxiety disorders	10 (4/6)	
Other psychiatric diagnosis	7 (6/1)	
No psychiatric diagnosis	158 (130/28)	
Unknown diagnosis	13 (6/7)	

*M* male, *F* female.

### Statistical Analysis

Statistical analyses were performed using the R software (https://www.r-project.org/). We calculated the frequency with which each somatic mosaic was detected as a function of age (<29, 30–39, 40–49, 50–59, 60–69, and ≥70). To investigate the association between autosomal mosaics and suicide death, we conducted logistic regression analyses. Each mosaic served as the objective variable, while suicide death, sample location, postmortem status, or postmortem interval (PMI) were considered as the explanatory variable as needed. Covariates [sex, age, age squared, and array for all analyses and smoking status as needed] were included in these analyses. Likewise, for sex chromosome mosaics, we performed logistic regression analyses to explore the association between each mosaic and suicide. Only females were analyzed for mLOX, and only males were analyzed for mLOY. Therefore, age, age squared, array for all subjects, and smoking status as needed, were considered as covariates in the analyses for mLOX and LOY. To integrate two cohorts (the first cohort and second cohort), we performed meta-analyses with fixed-effect models using the R package “metafor” (https://github.com/cran/metafor) [33]. The significance level was set at p < 0.05/6 ( = 8.33 × 10^−3^), using Bonferroni correction (six items: all autosomal mCAs, loss, CN-LOH, gain, mLOX, and mLOY).

For further investigation on the causal relationship between mLOX/mLOY and suicide death, we conducted two-sample Mendelian randomization (MR) analyses using the R package “TwoSampleMR” (https://github.com/MRCIEU/TwoSampleMR) [34]. SNPs were identified in discovery GWASs for mLOX [20] and mLOY [21], representing genetically determined risks for these conditions, and used as instrumental variables to investigate the causal effects of mLOX and mLOY on suicide death using summary statistics from our GWAS for suicide death [6] (Supplementary Table S2**)**. Meanwhile, no GWAS has identified genome-wide significant variants for suicide risk in an East Asian cohort, MR analyses to investigate the causal effects of suicide death on mLOX and mLOY were not conducted. Similarly, due to a lack of publicly available GWAS summary statistics for autosomal mosaicism, we did not conduct MR analyses to investigate the causal relationship between autosomal mosaicisms and suicide death.

## Results

### Autosomal chromosome mosaicisms in suicide

**Fig. 1:**
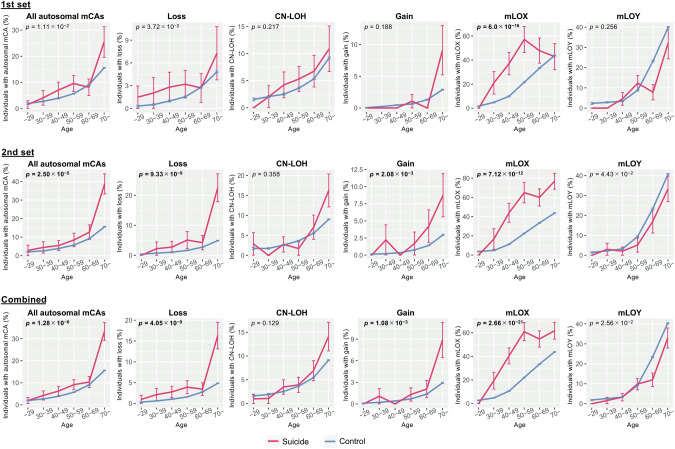
Frequency of detectable mosaicism stratified by age. The x- and y-axes represent the age category and percentage occurrence of each detectable mosaicism, respectively. Error bars indicate standard error. Blue lines denote the control group, and magenta lines denote the suicide decedent group. The p-values for the first and second sets were calculated using logistic regression analyses adjusted for sex (only for autosomal mosaicisms), age, age squared, and array. p-values for the meta-analysis were calculated using a fixed-effect model. CN-LOH copy-neutral loss of heterozygosity, mCA mosaic chromosomal alteration, mLOX mosaic loss of the X chromosome, mLOY mosaic loss of the Y chromosome.

**Fig. 2:**
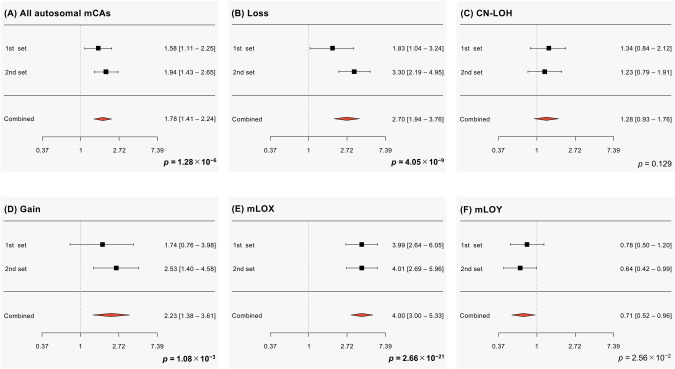
Forest plots of the odds ratios of suicide in detectable mosaicisms. Averages of the odds ratios and 95% confidence intervals of suicide in (**A**) all autosomal mCAs, (**B**) loss, (**C**) CN-LOH, (**D**) gain, (**E**) mLOX, and (**F**) mLOY are shown. p-values were calculated by meta-analyses using a fixed-effect model. CN-LOH copy-neutral loss of heterozygosity, mCA mosaic chromosomal alteration, mLOX mosaic loss of the X chromosome, mLOY mosaic loss of the Y chromosome.

The frequency of detectable autosomal mosaicism stratified by age in the suicide decedents and controls is shown in Fig. 1. In the first cohort, total mCAs and loss were nominally increased in suicide decedents compared to controls [all autosomal mCAs, p = 1.11 × 10^−2^, odds ratio (OR) = 1.58, 95%CI = 1.11–2.25; loss: p = 3.72×10^−2^, OR = 1.83, 95%CI = 1.04–3.24]. CN-LOHs and gain were not statistically associated with suicide [CN-LOH: p = 0.217, OR = 1.34, 95%CI = 0.84–2.12; gain: p = 0.188, OR = 1.74, 95%CI = 0.76–3.98]. In the second cohort, total mCAs, loss, and gain were again significantly increased in suicide decedents compared to controls (all autosomal mCAs, p = 2.50 × 10^−5^, OR = 1.94, 95%CI = 1.43–2.65; loss, p = 9.33 × 10^−9^, OR = 3.30, 95%CI = 2.19–4.95; gain, p = 2.08 × 10^−3^, OR = 2.53, 95%CI = 1.40–4.58). CN-LOHs were not statistically associated with suicide (p = 0.358, OR = 1.23, 95%CI = 0.79–1.91). Meta-analysis integrating the results from the first and second cohorts demonstrated that all autosomal mCAs, loss, and gain were significantly increased in suicide decedents compared to controls (all autosomal mCAs: p = 1.28 × 10^−6^, OR = 1.78, 95%CI = 1.41–2.24; loss: p = 4.05×10^−9^, OR = 2.70, 95%CI = 1.94–3.76; gain: p = 1.08×10^−3^, OR = 2.23, 95%CI = 1.38–3.61 (Fig. 2 and Supplementary Table S3). Notably, CN-LOHs, the most frequent type of mCA, were not significantly associated with suicide (p = 0.129, OR = 1.28, 95%CI = 0.93–1.76). The effects of all autosomal mCAs on suicide were in the same direction in the first and second sets (Fig. 2 and Supplementary Table S3).

The distribution of mCAs by autosomal chromosomes in suicide decedents and controls is shown in Fig. S3. The frequency of loss, CN-LOHs, and gain varied across chromosomal arms in a way that moderately correlated between suicide group and control group (R = 0.630, p = 1.45 × 10^−8^). Notably, loss of chromosome 14 was statistically more common in suicide decedents (p = 1.13 × 10^−4^) (Supplementary Table S4).

**Fig. 3:**
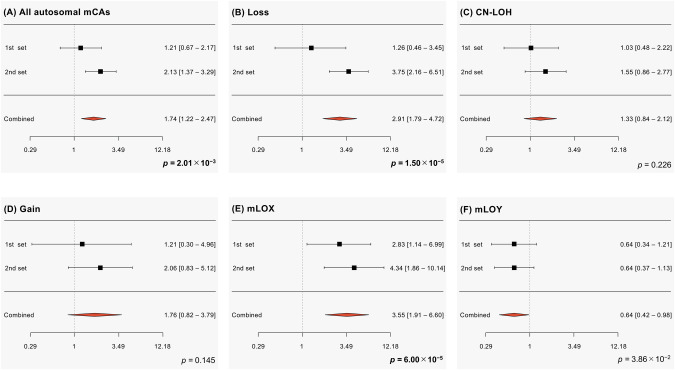
Forest plots of the odds ratios of suicide in detectable mosaicisms after excluding suicides with comorbid psychiatric disorders. Averages of the odds ratios and 95% confidence intervals of suicide in (**A**) all autosomal mCAs, (**B**) loss, (**C**) CN-LOH, (**D**) gain, (**E**) mLOX, and (**F**) mLOY are shown. p-values were calculated by meta-analyses using a fixed-effect model. CN-LOH copy-neutral loss of heterozygosity, mCA mosaic chromosomal alteration, mLOX mosaic loss of the X chromosome, mLOY mosaic loss of the Y chromosome.

To fix the potential confounding effects of comorbid psychiatric disorders on aberrant mosaicism, we conducted a subgroup analysis to test the association between autosomal mCAs and suicide death using only suicide decedents without diagnosed/possible psychiatric disorders (total suicides N = 303) and controls. We found associations with comparable effect sizes between mCAs and suicide decedents without psychiatric disorders (all autosomal mCAs: p = 2.01 × 10^−3^, OR = 1.74, 95%CI = 1.22–2.47; loss: p = 1.50 × 10^−5^, OR = 2.91, 95%CI = 1.79–4.72; CN-LOH: p = 0.226, OR = 1.33, 95%CI = 0.84–2.12; gain: p = 0.145, OR =1.76, 95%CI = 0.82–3.79), to those in the analysis using the whole sample of suicide decedents (Fig. 3, Fig. S4 and Supplementary Table S5).

### Sex chromosome mosaicisms in suicide

The frequency of X and Y chromosome loss stratified by age in the suicide decedents and control groups is shown in Fig. 1. In the first cohort, mLOX was significantly increased in female suicide decedents compared to female controls (p = 6.00 × 10^−16^, OR = 3.99, 95%CI = 2.64–6.05), while mLOY was not statistically associated with male suicide, but showed a rather protective association (p = 0.256, OR = 0.78, 95%CI = 0.50–1.20). Similarly, in the second cohort, mLOX was significantly increased in female suicide decedents compared to female controls (p = 7.12 × 10^−12^, OR = 4.01, 95%CI = 2.69–5.96), and mLOY was nominally associated with male suicide in a protective manner (p = 4.43 × 10^−2^, OR = 0.64, 95%CI = 0.42–0.99). Meta-analysis integrating the results from the first and second cohorts demonstrated that mLOX was significantly increased in female suicide decedents compared to female controls (p = 2.66 × 10^−21^, OR = 4.00, 95%CI = 3.00–5.33), while mLOY showed a trend of rather protective associations with male suicide decedents (p = 2.56 × 10^−2^, OR = 0.71, 95%CI = 0.52–0.96) (Fig. 2 and Supplementary Table S3). While the mean age of women was 49.55 years among the suicide decedents and 62.48 years among the controls, mLOX was observed in 110 out of 258 (42.6%) suicide decedents and 24,896 of 82,447 (30.2%) controls. The effects of mLOX and mLOY on suicide were in the same direction in the first and second sets (Fig. 2 and Supplementary Table S3).

We also conducted a subgroup analysis to test the association between mLOX/mLOY and suicide deaths using sex-specific suicide decedents without diagnosed/possible psychiatric disorders (total male and female suicides were 247 and 56, respectively) and sex-specific controls. We found associations with comparable effect sizes between mLOX and female suicide decedents without psychiatric disorders (mLOX, p = 6.00×10^−5^, OR = 3.55, 95%CI = 1.91–6.60), to those in the analysis using the whole sample of suicide decedents (Fig. 3, Fig. S4 and Supplementary Table S5).

We conducted MR analysis to test the effects of genetically determined risk of mLOX and mLOY on suicide death using genome-wide significant SNPs identified in discovery GWASs for mLOX and mLOY. Subsequently, no evidence was found for the effect of genetically determined risk of mLOX and mLOY on suicide death (Fig. S5 and Supplementary Table S2).

### No effects of potential confounding factors on aberrant mCAs in suicide death

To address the potential confounding effects of comorbid physical diseases on aberrant mosaicism, we conducted a subgroup analysis to test the association between mCAs and suicide death using only suicide decedents without severe physical diseases (total suicides N = 637) and controls. We found similar associations between mCAs and suicide decedents without severe physical diseases compared with the analysis using the whole sample of suicide decedents (Supplementary Table S6).

To account for the potential confounding effects of smoking status on aberrant mosaicism, we conducted a subgroup analysis to test the association between mCAs and suicide death with smoking status as an additional covariate, using only subjects with accurate smoking status information (total suicides N = 711 and controls N = 175,190). We found similar associations between mCAs and suicide decedents when smoking status was considered, similar to those in the whole sample analysis (Supplementary Table S7).

To test the potential confounding effects of the different sample locations (suicide cases from the local Kobe University laboratory, main BBJ controls from all over Japan), we also conducted an mCA analysis between BBJ controls and healthy participants collected at the Kobe University laboratory and confirmed that autosomal mCAs and mLOX were not increased in the local healthy participants compared to BBJ controls (Supplementary Table S8).

To test the potential confounding effects of the different biological statuses of samples (blood from postmortem suicide cases vs. from living controls), we conducted an mCA analysis between the postmortem suicide decedents and postmortem non-suicide decedents collected at the same Kobe University laboratory and found similar association patterns as those in the comparison between postmortem suicide decedents and the living controls from BBJ (Supplementary Table S9). We also conducted an mCA analysis between the postmortem non-suicide decedents collected at the Kobe University laboratory and the controls from BBJ and confirmed that autosomal mCAs and mLOX were not increased in the postmortem non-suicide decedents compared to BBJ controls (Supplementary Table S10). In addition, we found no significant associations between any mCAs and PMI (Supplementary Table S11).

The distribution of individuals by the number of mCA events in suicide decedents and controls is shown in Supplementary Table S12. To ensure that the above-mentioned results are not biased by outliers with very large numbers of autosomal mCA events, we conducted a subgroup analysis using only suicide decedents and controls with fewer than ten mCA events (772 suicide decedents and 177,735 controls). This analysis showed similar associations with effect sizes between mCAs and suicide decedents comparable to those in the whole-sample analyses (Supplementary Table S13).

## Discussion

To the best of our knowledge, this is the first study to examine the variations in somatic mosaics identified in genome-wide genotyping data within suicide decedents and controls. The results from our integrated meta-analyses of two independent cohorts revealed a considerable increase in all autosomal mCAs, loss and gain in autosomal chromosomes, and mLOX in sex chromosomes among suicide deaths. These associations fulfilled a stringent statistical significance based on Bonferroni’s correction. No statistically significant difference was observed in autosomal CN-LOHs and mLOY between suicide deaths and controls. mLOY showed a trend of rather protective associations with suicide. Our in-depth subgroup analyses support the notion that these aberrant mCAs are due mostly to suicide death itself and not confounded by comorbid psychiatric disorders or physical diseases, smoking status, sample location, postmortem sample status, or outliers with very large numbers of autosomal mCA events.

While several previous studies have shown aberrant chromosome Y alterations in suicide-related psychiatric traits by quantitative fluorescence polymerase chain reaction (QF-PCR) and TaqMan assays based on homologous amelogenin genes [18, 35], to the best of our knowledge, no study has revealed aberrant mCAs in any suicidal phenotype (suicidal ideation, non-fatal suicidal behavior, and suicide death) by leveraging SNP-array data (MoChA).

Previous studies have indicated that these mCAs are linked to aging, which suggests that an aberrant increase in mCAs in suicide deaths may be associated with abnormal aging in suicide decedents [15, 36, 37]. Consistent with this hypothesis, several studies found replicable results regarding accelerated biological aging in individuals attempting suicide and suicide decedents, such as shortened telomere length and accelerated epigenetic clock aging [9–12]. Given that the past literature had shown that shortened telomere length and accelerated epigenetic clock aging mainly seem to be the consequences of excessive mental stress [38, 39], we consider that the increased mCAs demonstrated here may also mainly be the consequences of suicide, which represents one of the most extreme phenotype of mental stress. Further studies focusing on mCAs and biological aging indicators (e.g., telomeres and epigenetic clocks) in the same individuals may enable the elucidation of the biological pathway causing aberrant age acceleration in suicide risk.

In this study, we demonstrated a significantly increased mosaic loss for the X chromosome, even more than autosomal mosaicism. While no study has investigated the association between mLOX and suicidal behavior and suicide-related psychiatric traits, several X-linked disorders are known to link with psychiatric traits. For instance, fragile X syndrome, an X-linked neurodevelopmental disorder in which the fragile X messenger ribonucleoprotein, located at Xq27.3, is a well-known contributor to autism spectrum disorders, mental retardation, anorexia, social withdrawal, and depressive symptoms [40–44]. In addition, other X-linked disorders such as Klinefelter syndrome [45, 46] and Turner’s syndrome [47–49] are also known to increase the risk of anxiety, impulsivity, attention deficit disorder, major depression, autism spectrum disorder, and schizophrenia. However, no study exists on the role of mLOX in suicidal phenotype. Our findings further our understanding of the link between X-chromosome dysfunction and suicide risk. In this study, we found that mLOY showed a trend of negative association with suicide. While no study has investigated the association between mLOY and suicidal behavior by leveraging blood-derived SNP-array data, our small study (130 suicide cases) previously indicated the existence of a subpopulation of suicide decedents with aberrantly increased blood mLOY using the QF-PCR method [50], which contradicts the results of the current study. Given the larger sample sizes and more established SNP array data used in the current study, we withhold our past insights regarding the association between aberrantly increased mLOY and suicide.

Our study has several limitations. First, although our sample size for suicide GWAS is the largest ever used for studying suicide deaths in the East Asian population, larger sample sizes would be preferable for SNP array-based research. Second, our study cohort was restricted to the East Asian population; thus, the findings from the present study need to be tested in other populations. Third, the connection between increased mCAs and suicide may be exaggerated due to various potential confounding impacts (e.g. comorbid psychiatric disorders and physical diseases, smoking status, sample location, and postmortem sample status). In fact, several studies have indicated aberrant mCAs in specific psychiatric disorders and physical diseases such as bipolar disorder, schizophrenia, cancer, and cardiovascular disease [23, 27, 51]. Several studies have revealed that smoking status similarly affects mCAs [15] and that smoking status is epidemiologically and genetically associated with suicidal behavior [4, 52]. In addition, our main cohort (suicide decedents vs. BBJ controls) involved the disparities in sample location and biological status of samples (suicide case used postmortem blood samples from the local Kobe University laboratory, whereas controls were from all over Japan whose blood samples were taken when they were alive), which may affect mCAs. In this study, we conducted several in-depth subgroup analyses to fix these potential confounding effects on aberrant mCAs. We conducted 1) subgroup analysis after excluding suicide decedents with diagnosed/possible psychiatric disorders and comorbid severe physical diseases and 2) subgroup analysis using only subjects with accurate smoking status information, adjusting for smoking status as a covariate. We identified similar association patterns with comparable effect sizes between mCAs and suicide deaths in both analyses, consistent with those found using the whole sample of suicide decedents and controls. Furthermore, using an additional supplementary cohort comprising samples from healthy participants and non-suicide decedents collected at the local Kobe University laboratory, we confirmed no increase in autosomal mCAs and mLOX when comparing alive/postmortem controls from Kobe University with BBJ controls. These suggest that our findings were mostly driven by suicide itself, and not confounded by comorbid psychiatric disorders or physical diseases, smoking status, sample location, or postmortem sample status. Nevertheless, SNP array-based research to precisely examine the effects of aberrant mCAs on suicide risk, independent of these potential confounders, requires a larger sample size given that each of our subgroup analyses and the supplementary cohort had limitations terms of their small sample size. In addition, to precisely estimate whether mCAs may be biased by postmortem sample status, future investigation using biological samples obtained both pre- and postmortem from the same individual would be needed. Fourth, we could not exclude other potential confounders (e.g. body mass index) that are documented to affect mCAs as well [18]. Fifth, the subjects used as non-suicide controls were not screened psychiatrically. In addition, most controls had various non-psychiatric disorders. However, this approach has already been applied to previous GWASs, the results of which were replicable in other recent GWAS [53, 54], supporting the reliability of our results. Sixth, the biological interpretation of aberrant mCAs in suicide deaths reported here remains largely unknown because of the lack of evidence from similar past studies. For instance, while we detected statistically significant loss of chromosome 14 in suicide decedents, no past literature showed evidence of association between variants or epigenetic changes on chromosome 14 and suicidal phenotype. Although we conducted MR analysis to test the effects of genetically determined risk of mLOX and mLOY on suicide death, we did not find evidence for an effect. Due to lack of GWAS identifying genome-wide significant variants for suicide risk in an East Asian cohort and available GWAS summary statistics of autosomal mCAs for MR analysis, we could not investigate the effect of genetically determined risk of mLOX/mLOY on suicide death or any causal relationship between autosomal mosaicisms and suicide death. Therefore, future prospective studies using larger sample sizes of suicide GWASs and GWASs of autosomal mCAs are needed to more precisely determine the causal relationship between mCAs and suicide.

In conclusion, we provide the first evidence of aberrant mosaic alterations in somatic autosomal and sex chromosomes in suicide deaths, and our results may contribute to an improved understanding of the genomic pathophysiology underlying suicide.

## Supplementary information

Supplementary Tables and Figures

## Data Availability

All computational codes are available upon request from the corresponding authors (although they are not immediately portable to other computing environments). A standalone software implementation (MoChA) of the algorithm used to call mCAs is available at https://github.com/freeseek/mocha.
