# Supplementary material for: Increased somatic mosaicism in autosomal and X chromosomes for suicide death
Source: Mol Psychiatry. 2024 Aug 30;30(3):881–8. doi: 10.1038/s41380-024-02718-y (PMC11835753; doi:10.1038/s41380-024-02718-y)
Supplement: Supplementary file 1 — Supplementary Tables and Figures [file 41380_2024_2718_MOESM1_ESM.pdf]

Increased somatic mosaicism in autosomal and X chromosome for suicide death

Supplementary Tables

S1. Demographic data of healthy participants and non-suicide decedents as a supplementary cohort
S2. Detailed information on two-sample Mendelian Randomization analysis
S3. The results of logistic regression analyses and meta-analysis for mCAs in suicide death
S4. Distribution of autosomal mCAs in suicide decedents and controls
S5. The results of logistic regression analyses and meta-analysis for mCAs in suicide death after excluding suicide decedents with diagnosed/possible psychiatric disorders
S6. The results of logistic regression analyses and meta-analysis for mCAs in suicide death after excluding suicide decedents with comorbid severe physical diseases
S7. The results of logistic regression analyses and meta-analysis for mCAs in suicide death adjusting for smoking status as an additional covariate
S8. The results of logistic regression analyses for mCAs between Kobe healthy participants and BBJ controls
S9. The results of logistic regression analyses for mCAs between suicide decedents and non-suicide decedents
S10. The results of logistic regression analyses for mCAs in non-suicide decedents and BBJ controls
S11. The results of logistic regression analyses between mCAs and postmortem interval
S12. Distribution of mCA events in suicide decedents and controls
S13. The results of logistic regression analyses and meta-analysis for mCAs in suicide death using only subjects with fewer than ten mCA events

Supplementary Figures

S1. The results of principal component analysis (PCA)
S2. An overview of the study cohort

S3. Distribution of detectable mosaicisms stratified by each autosomal chromosome

S4. Frequency of detectable mosaicism stratified by age, after excluding suicide decedents with diagnosed/possible psychiatric disorders

S5. Scatter plot of MR analysis inferring causal effect of mLOX and mLOY on suicide death

Table S1. Demographic data of healthy participants and non-suicide decedents as a supplementary cohort

	Healthy participants [a]	Non-suicide decedents [a]
Number of subjects (M/F)	138 (77/61)	91 (53/38)
Age (mean ± standard deviation, years)	M: 45.27 ± 16.53	M: 48.00 ± 14.75
	F: 46.79 ± 21.36	F: 52.34 ± 14.85
Array		
Illumina Human OmniExpressExome v1.4	138	91
Cause of death		
Cardiovascular disease		37 (25/12)
Stroke		11 (6/5)
Traffic accident		9 (6/3)
Endocrine disease		6 (2/4)
Drowning		4 (2/2)
Intestinal disease		4 (1/3)
Pulmonary disease		4 (2/2)
Sepsis		4 (2/2)
Frozen		3 (1/2)
Suffocation		3 (1/2)
Epilepsy		2 (2/0)
Liver disease		2 (1/1)
Falling		1 (1/0)
Impalement injury		1 (1/0)

[a] DNA samples of healthy participants were extracted from blood when they were alive, and those of non-suicide decedents were extracted from postmortem blood after death (not due to suicide). All sampling and DNA extraction were performed in the same the Department of Psychiatry and Division of Legal Medicine at Kobe University Graduate School of Medicine between 2010 and 2024.

M: male, F: female

Table S2. Detailed information on two-sample Mendelian Randomization analysis

GWAS summary statistics and instrument variables

Exposure	Outcome	GWAS	Ethnicity	Number of identified SNPs in exposure GWAS	Number of SNPs used as instrument variables [a]
mLOX	Suicide	Liu et al. 2024 [1] (exposure)	Trans-ethnic [b]	56	29
		Otsuka et al. 2019 [2] (outcome)	Japanese		
mLOY	Suicide	Terao et al. 2019 [3] (exposure)	Japanese	92 [c]	88
		Otsuka et al. 2019 [2] (outcome)	Japanese		

MR analyses inferring causality of mLOX/mLOY on suicide

Exposure	Outcome	MR-Method	Beta (SE)	p value
mLOX	Suicide	IVW	−0.046 (0.210)	0.825
		MR-Egger	−0.319 (0.379)	0.407
		Simple median	−0.089 (0.396)	0.823
		Weighted median	−0.303 (0.306)	0.322
mLOY	Suicide	IVW	−0.032 (0.251)	0.900
		MR-Egger	0.314 (0.433)	0.470
		Simple median	−0.390 (0.447)	0.383
		Weighted median	0.006 (0.374)	0.987

Exposure	Outcome	Egger intercept (SE)	p value
mLOX	Suicide	0.019 (0.022)	0.394
mLOY	Suicide	−0.013 (0.013)	0.330

[a] Among the identified SNPs in exposure GWAS, only these SNPs were included in outcome GWAS for suicide [2].
[b] This GWAS [1] included Europeans and Japanese.
[c] These SNPs were significant ($P < 5\times10^{-8}$) in European mLOY GWAS (Thompson et al. *Nature* 2019 [4]) and also in the same direction of effects in Japanese mLOY GWAS (Terao et al. 2019 [3]).

GWAS, genome-wide association study; IVW, inverse variance weighted; mLOX, mosaic loss of the X chromosome; mLOY, mosaic loss of the Y chromosome; MR, Mendelian randomization; No., number; SE, standard error; SNP, Single nucleotide polymorphism

REFERENCES

1. Liu A, Genovese G, Zhao Y, Pirinen M, Zekavat SM, Kentistou KA *et al.* Genetic drivers and cellular selection of female mosaic X chromosome loss. *Nature* 2024; Epub ahead of print.
2. Otsuka I, Akiyama M, Shirakawa O, Okazaki S, Momozawa Y, Kamatani Y *et al.* Genome-wide association studies identify polygenic effects for completed suicide in the Japanese population. *Neuropsychopharmacology* 2019; 44: 2119–2124.
3. Terao C, Momozawa Y, Ishigaki K, Kawakami E, Akiyama M, Loh PR *et al.* GWAS of mosaic loss of chromosome Y highlights genetic effects on blood cell differentiation. *Nat Commun* 2019; 10: 4719.
4. Thompson DJ, Genovese G, Halvardson J, Ulirsch JC, Wright DJ, Terao C *et al.* Genetic predisposition to mosaic Y chromosome loss in blood. *Nature* 2019; 575: 652–657.

Table S3. The results of logistic regression analyses and meta-analysis for mCAs in suicide death

	Number of subjects with mCA events/ number of subjects		OR (95% CI)	p value	P_{het} [a]
All autosomal mCAs [b]	Suicide decedents	Controls			
1st set	37/410	8,731/88,870	1.58 (1.11 – 2.25)	1.11×10^{-2} [c]	
2nd set	52/363	8,814/88,870	1.94 (1.43 – 2.65)	**2.50×10^{-5}** [c]	
Combined			1.78 (1.41 – 2.24)	**1.28×10^{-6}** [d]	0.388
Loss	Suicide decedents	Controls			
1st set	13/410	2,636/88,870	1.83 (1.04 – 3.24)	3.72×10^{-2} [c]	
2nd set	27/363	2,667/88,870	3.30 (2.19 – 4.95)	**9.33×10^{-9}** [c]	
Combined			2.70 (1.94 – 3.76)	**4.05×10^{-9}** [d]	0.100
CN-LOH	Suicide decedents	Controls			
1st set	20/410	5,270/88,870	1.34 (0.84 – 2.12)	0.217 [c]	
2nd set	22/363	5,237/88,870	1.23 (0.79 – 1.91)	0.358 [c]	
Combined			1.28 (0.93 – 1.76)	0.129 [d]	0.797
Gain	Suicide decedents	Controls			
1st set	6/410	1,417/88,870	1.74 (0.76 – 3.98)	0.188 [c]	
2nd set	12/363	1,485/88,870	2.53 (1.40 – 4.58)	**2.08×10^{-3}** [c]	
Combined			2.23 (1.38 – 3.61)	**1.08×10^{-3}** [d]	0.471
mLOX	Suicide decedents	Controls			
1st set	46/135	12,367/41,223	3.99 (2.64 – 6.05)	**6.00×10^{-16}** [c]	
2nd set	64/123	12,529/41,224	4.01 (2.69 – 5.96)	**7.12×10^{-12}** [c]	
Combined			4.00 (3.00 – 5.33)	**2.66×10^{-21}** [d]	0.992
mLOY	Suicide decedents	Controls			
1st set	26/275	11,290/47,647	0.77 (0.50 – 1.20)	0.256 [c]	
2nd set	29/240	11,445/47,646	0.64 (0.42 – 0.99)	4.43×10^{-2} [c]	
Combined			0.71 (0.52 – 0.96)	2.56×10^{-2} [d]	0.552

[a] P for heterogeneity between two analyses (first set and second set) were calculated by Cochran's Q test.
[b] This indicates the number of subjects with at least one of loss, CN-LOH or gain in autosomal chromosomes.

[c] The p values were calculated with logistic regression analyses, using sex, age and age-squared as confounding factors.
[d] The p values were calculated with meta-analyses with the fixed-effect model. The p values in bold indicate statistically significant ($p < 0.05/6$ [$= 8.33\times10^{-3}$]).

CI, confidence interval; CN-LOH, copy-neutral loss of heterozygosity; mCAs, mosaic chromosomal alterations; mLOX, mosaic loss of the X chromosome; mLOY, mosaic loss of the Y chromosome; OR, odds ratio

Table S4. Distribution of autosomal mCAs in suicide decedents and controls

Chromosome	Loss			CN-LOH			Gain		
	Suicide decedents	Controls	p value [a]	Suicide decedents	Controls	p value [a]	Suicide decedents	Controls	p value [a]
1	0.83%	1.86%	0.729	1.67%	7.80%	$8.89×10^{-3}$*	0.83%	0.09%	0.102
2	1.67%	1.31%	0.673	3.33%	1.89%	0.294	1.67%	0.06%	$3.07×10^{-3}$*
3	1.67%	0.99%	0.334	3.33%	1.60%	0.129	1.67%	0.29%	0.0485*
4	2.50%	1.22%	0.182	1.67%	1.18%	0.655	0.00%	0.16%	1.000
5	2.50%	1.65%	0.453	0.83%	0.98%	1.000	0.00%	0.03%	1.000
6	0.00%	1.88%	0.178	3.33%	2.96%	0.783	0.00%	0.06%	1.000
7	1.67%	1.24%	0.662	0.00%	1.09%	0.643	0.00%	0.03%	1.000
8	2.50%	0.77%	0.0669	0.83%	1.18%	1.000	1.67%	1.49%	0.700
9	0.00%	1.14%	0.649	3.33%	3.43%	1.000	1.67%	0.54%	0.140
10	0.83%	0.46%	0.428	0.83%	1.03%	1.000	0.00%	0.03%	1.000
11	1.67%	2.29%	1.000	2.50%	4.35%	0.496	0.00%	0.05%	1.000
12	0.00%	0.64%	1.000	0.83%	1.48%	1.000	0.83%	0.39%	0.378
13	1.67%	1.96%	1.000	1.67%	1.56%	0.712	0.00%	0.25%	1.000
14	10.00%	2.69%	$1.13×10^{-4}$**	2.50%	10.22%	$2.21×10^{-3}$*	2.50%	1.38%	0.231
15	0.00%	0.59%	1.000	1.67%	2.41%	1.000	1.67%	3.74%	0.332
16	0.83%	1.00%	1.000	3.33%	2.35%	0.371	0.00%	0.03%	1.000
17	0.00%	1.17%	0.653	0.83%	2.42%	0.375	0.83%	0.07%	0.0859
18	1.67%	0.43%	0.0972	1.67%	0.71%	0.210	0.00%	0.69%	1.000
19	0.83%	0.38%	0.370	0.00%	1.68%	0.273	0.00%	0.05%	1.000
20	4.17%	4.15%	1.000	1.67%	1.44%	0.693	0.83%	0.07%	0.0859
21	1.67%	0.54%	0.140	1.67%	2.13%	1.000	5.00%	4.00%	0.485
22	1.67%	0.88%	0.287	0.83%	1.86%	0.729	4.17%	1.58%	0.0435*

This table provides numeric data plotted in Fig. S2. Frequencies indicate the contribution of each event type to the total number of mCAs classified as loss, CN-LOH, or gain in each data set.

[a] p values were calculated with Fisher's exact test. p values in bold indicate $p < 0.05$.

* nominally significant ($p < 0.05$), ** significant after Bonferroni adjustment ($p < 0.05/66$ [= $7.58×10^{-4}$])

CN-LOH, copy-neutral loss of heterozygosity; mCAs, mosaic chromosomal alterations

Table S5. The results of logistic regression analyses and meta-analysis for mCAs in suicide death after excluding suicide decedents with diagnosed/possible psychiatric disorders

	Number of subjects with mCA events/ number of subjects		OR (95% CI)	p value	P_{het} [a]
All autosomal mCAs [b]	Suicide decedents (no psychiatric disorders)	Controls			
1st set	13/145	8,731/88,870	1.21 (0.67 – 2.17)	0.527 [c]	
2nd set	27/158	8,814/88,870	2.13 (1.37 – 3.29)	**7.14×10^{-4}** [c]	
Combined			1.74 (1.22 – 2.47)	**2.01×10^{-3}** [d]	0.130
Loss	Suicide decedents	Controls			
1st set	4/145	2,636/88,870	1.26 (0.46 – 3.45)	0.648 [c]	
2nd set	15/158	2,667/88,870	3.75 (2.16 – 6.51)	**2.73×10^{-6}** [c]	
Combined			2.91 (1.79 – 4.72)	**1.50×10^{-5}** [d]	0.0636
CN-LOH	Suicide decedents	Controls			
1st set	7/145	5,270/88,870	1.03 (0.48 – 2.22)	0.946 [c]	
2nd set	13/158	5,237/88,870	1.55 (0.86 – 2.77)	0.142 [c]	
Combined			1.33 (0.84 – 2.12)	0.226 [d]	0.406
Gain	Suicide decedents	Controls			
1st set	2/145	1,417/88,870	1.21 (0.30 – 4.96)	0.789 [c]	
2nd set	5/158	1,485/88,870	2.06 (0.83 – 5.12)	0.118 [c]	
Combined			1.76 (0.82 – 3.79)	0.145 [d]	0.535
mLOX	Suicide decedents	Controls			
1st set	10/28	12,367/41,223	2.83 (1.14 – 6.98)	**2.45×10^{-2}** [c]	
2nd set	16/28	12,529/41,224	4.34 (1.86 – 10.14)	**6.86×10^{-4}** [c]	
Combined			3.55 (1.91 – 6.60)	**6.00×10^{-5}** [d]	0.497
mLOY	Suicide decedents	Controls			
1st set	12/117	11,290/47,647	0.64 (0.34 – 1.21)	0.169 [c]	
2nd set	17/130	11,445/47,646	0.64 (0.37 – 1.13)	0.122 [c]	
Combined			0.64 (0.42 – 0.98)	3.86×10^{-2} [d]	0.985

[a] P for heterogeneity between two analyses (first set and second set) were calculated by Cochran's Q test.
[b] This indicates the number of subjects with at least one of loss, CN-LOH or gain in autosomal chromosomes.
[c] The p values were calculated with logistic regression analyses, using sex, age and age-squared as confounding factors.
[d] The p values were calculated with meta-analyses with the fixed-effect model. The p values in bold indicate statistically significant ($p < 0.05/6$ [= 8.33×10^{-3}]).

CI, confidence interval; CN-LOH, copy-neutral loss of heterozygosity; mCAs, mosaic chromosomal alterations; mLOX, mosaic loss of the X chromosome; mLOY, mosaic loss of the Y chromosome; OR, odds ratio

Table S6. The results of logistic regression analyses and meta-analysis for mCAs in suicide death after excluding suicide decedents with comorbid severe physical diseases [a]

	Number of subjects with mCA events/ number of subjects		OR (95% CI)	p value	P_{het} [b]
All autosomal mCAs [c]	Suicide decedents (no physical diseases)	Controls			
1st set	29/344	8,731/88,870	1.53 (1.04 – 2.27)	0.0323 [d]	
2nd set	35/293	8,814/88,870	1.97 (1.37 – 2.85)	$\mathbf{2.87\times10^{-4}}$ [d]	
Combined			1.75 (1.34 – 2.29)	$\mathbf{4.20\times10^{-5}}$ [e]	0.357
Loss	Suicide decedents	Controls			
1st set	12/344	2,636/88,870	2.27 (1.26 – 4.07)	$\mathbf{6.30\times10^{-3}}$ [d]	
2nd set	16/293	2,667/88,870	2.96 (1.77 – 4.97)	$\mathbf{3.93\times10^{-5}}$ [d]	
Combined			2.64 (1.79 – 3.89)	$\mathbf{9.31\times10^{-7}}$ [e]	0.495
CN-LOH	Suicide decedents	Controls			
1st set	15/344	5,270/88,870	1.2 (0.71 – 2.03)	0.488 [d]	
2nd set	17/293	5,237/88,870	1.45 (0.88 – 2.39)	0.143 [d]	
Combined			1.33 (0.93 – 1.91)	0.124 [e]	0.613
Gain	Suicide decedents	Controls			
1st set	4/344	1,417/88,870	1.56 (0.58 – 4.23)	0.381 [d]	
2nd set	6/293	1,485/88,870	2.13 (0.93 – 4.84)	0.0724 [d]	
Combined			1.88 (1.00 – 3.54)	0.0518 [e]	0.639
mLOX	Suicide decedents	Controls			
1st set	37/119	12,367/41,223	2.72 (1.73 – 4.26)	$\mathbf{1.35\times10^{-5}}$ [d]	
2nd set	51/105	12,529/41,224	4.25 (2.77 – 6.53)	$\mathbf{3.54\times10^{-11}}$ [d]	
Combined			3.44 (2.52 – 4.70)	$\mathbf{6.54\times10^{-15}}$ [e]	0.157
mLOY	Suicide decedents	Controls			
1st set	18/225	11,290/47,647	0.76 (0.45 – 1.27)	0.292 [d]	
2nd set	15/188	11,445/47,646	0.58 (0.33 – 1.03)	6.12×10^{-2} [d]	
Combined			0.67 (0.46 – 0.99)	4.22×10^{-2} [e]	0.0423

[a] This subgroup analyses included suicide decedents whose comorbid severe physical diseases were considered to be the cause of suicide, as determined through psychological autopsies based on their medical records and interviews with their bereaved family members.
[b] P for heterogeneity between two analyses (first set and second set) were calculated by Cochran's Q test.
[c] This indicates the number of subjects with at least one of loss, CN-LOH or gain in autosomal chromosomes.
[d] The p values were calculated with logistic regression analyses, using sex, age and age-squared as confounding factors.
[e] The p values were calculated with meta-analyses with the fixed-effect model. The p values in bold indicate statistically significant ($p < 0.05/6$ [$= 8.33\times10^{-3}$]).

CI, confidence interval; CN-LOH, copy-neutral loss of heterozygosity; mCAs, mosaic chromosomal alterations; mLOX, mosaic loss of the X chromosome; mLOY, mosaic loss of the Y chromosome; OR, odds ratio

Table S7. The results of logistic regression analyses and meta-analysis for mCAs in suicide death adjusting for smoking status as an additional covariate [a]

	Number of subjects with mCA events/ number of subjects		OR (95% CI)	p value	P het [b]
All autosomal mCAs [c]	Suicide decedents (with smoking status)	Controls (with smoking status)			
1st set	33/382	8,571/87,600	1.40 (0.97 – 2.03)	0.0710 [d]	
2nd set	50/329	8,675/87,590	2.19 (1.60 – 3.01)	**1.11×10^{-6}** [d]	
Combined			1.82 (1.43 – 2.31)	**1.10×10^{-6}** [e]	0.0717
Loss	Suicide decedents	Controls			
1st set	12/382	2,579/87,600	1.75 (0.98 – 3.15)	0.0604 [d]	
2nd set	26/329	2,625/87,590	3.62 (2.39 – 5.49)	**1.32×10^{-9}** [d]	
Combined			2.84 (2.02 – 3.99)	**1.62×10^{-9}** [e]	0.0476
CN-LOH	Suicide decedents	Controls			
1st set	17/382	5,187/87,600	1.13 (0.69 – 1.85)	0.631 [d]	
2nd set	20/329	5,155/87,590	1.32 (0.83 – 2.09)	0.239 [d]	
Combined			1.23 (0.88 – 1.72)	0.235 [e]	0.652
Gain	Suicide decedents	Controls			
1st set	5/382	1,387/87,600	1.42 (0.58 – 3.48)	0.439 [d]	
2nd set	11/329	1,463/87,590	2.58 (1.39 – 4.79)	**2.68×10^{-3}** [d]	
Combined			2.13 (1.28 – 3.54)	**3.60×10^{-3}** [e]	0.284
mLOX	Suicide decedents	Controls			
1st set	43/126	12,177/40,613	2.88 (1.88 – 4.42)	**1.25×10^{-6}** [d]	
2nd set	60/107	12,342/40,599	5.12 (3.33 – 7.88)	**1.15×10^{-13}** [d]	
Combined			3.84 (2.83 – 5.20)	**4.24×10^{-18}** [e]	0.0641
mLOY	Suicide decedents	Controls			
1st set	25/256	11,112/46,987	0.80 (0.51 – 1.24)	0.317 [d]	
2nd set	29/222	11,269/46,991	0.84 (0.55 – 1.30)	0.442 [d]	
Combined			0.82 (0.60 – 1.12)	0.212 [e]	0.854

[a] In this subgroup analyses, suicide decedents and controls with accurate information on smoking status were included. The first cohort consisted of 382 suicide decedents (140 smokers and 142 non-smokers) and 87,600 controls (43,788 smokers and 43,812 non-smokers), and the second cohort consisted of 329 suicide decedents (130 smokers and 199 non-smokers) and 87,590 controls (43,812 smokers and 43,668 non-smokers).
[b] P for heterogeneity between two analyses (first set and second set) were calculated by Cochran's Q test.
[c] This indicates the number of subjects with at least one of loss, CN-LOH or gain in autosomal chromosomes.
[d] The p values were calculated with logistic regression analyses, using sex, age, age-squared and smoking status as confounding factors.
[e] The p values were calculated with meta-analyses with the fixed-effect model. The p values in bold indicate statistically significant ($p < 0.05/6$ [$= 8.33\times10^{-3}$]).

CI, confidence interval; CN-LOH, copy-neutral loss of heterozygosity; mCAs, mosaic chromosomal alterations; mLOX, mosaic loss of the X chromosome; mLOY, mosaic loss of the Y chromosome; OR, odds ratio

Table S8. The results of logistic regression analyses for mCAs between Kobe healthy participants and BBJ controls

	Number of subjects with mCA events/ number of subjects		OR (95% CI)	p value [a]
	Kobe healthy participants [b]	BBJ controls [b]		
All autosomal mCAs [c]	5/138	17,545/177,740	0.62 (0.25 – 1.53)	0.298
Loss	3/138	5,303/177,740	1.38 (0.43 – 4.40)	0.582
CN-LOH	3/138	10,507/177,740	0.59 (0.19 – 1.88)	0.377
Gain	2/138	2,902/177,740	1.93 (0.47 – 7.94)	0.361
mLOX	5/61	24,896/82,447	0.37 (0.14 – 0.96)	0.0421
mLOY	5/77	22,735/95,293	0.63 (0.24 – 1.65)	0.343

[a] The p values were calculated with logistic regression analyses, using sex, age and age-squared as confounding factors. The p values in bold indicate statistically significant ($p < 0.05/6$ [$= 8.33\times10^{-3}$]).
[b] Kobe healthy participants were collected at the local Kobe University laboratory, whereas main BBJ controls from all over Japan.
[c] This indicates the number of subjects with at least one of loss, CN-LOH or gain in autosomal chromosomes.

BBJ, BioBank Japan; CI, confidence interval; CN-LOH, copy-neutral loss of heterozygosity; mCAs, mosaic chromosomal alterations; mLOX, mosaic loss of the X chromosome; mLOY, mosaic loss of the Y chromosome; OR, odds ratio

Table S9. The results of logistic regression analyses for mCAs between suicide decedents and non-suicide decedents

	Number of subjects with mCA events/ number of subjects		OR (95% CI)	p value [a]
	Suicide decedents [b]	Non-Suicide decedents [b]		
All autosomal mCAs [c]	89/773	5/91	1.77 (0.68 – 4.63)	0.242
Loss	40/773	1/91	3.88 (0.52 – 29.01)	0.187
CN-LOH	42/773	3/91	1.30 (0.38 – 4.38)	0.675
Gain	18/773	1/91	1.47 (0.18 – 11.68)	0.716
mLOX	110/258	5/38	6.03 (2.21 – 16.46)	**4.53×10^{-4}**
mLOY	55/515	5/53	0.75 (0.27 – 2.11)	0.589

[a] The p values were calculated with logistic regression analyses, using sex, age and age-squared as confounding factors. The p values in bold indicate statistically significant ($p < 0.05/6$ [= 8.33×10^{-3}]).
[b] Suicide decedents and non-suicide decedents were collected at the local Kobe University laboratory.
[c] This indicates the number of subjects with at least one of loss, CN-LOH or gain in autosomal chromosomes.

CI, confidence interval; CN-LOH, copy-neutral loss of heterozygosity; mCAs, mosaic chromosomal alterations; mLOX, mosaic loss of the X chromosome; mLOY, mosaic loss of the Y chromosome; OR, odds ratio

Table S10. The results of logistic regression analyses for mCAs in non-suicide decedents and BBJ controls

	Number of subjects with mCA events/ number of subjects		OR (95% CI)	p value [b]
	Non-suicide decedents	BBJ controls		
All autosomal mCAs [a]	5/91	17,545/177,740	0.93 (0.37 – 2.32)	0.875
Loss	1/91	5,303/177,740	0.66 (0.09 – 4.74)	0.675
CN-LOH	3/91	10,507/177,740	0.89 (0.28 – 2.82)	0.836
Gain	1/91	2,902/177,740	1.42 (0.20 – 10.33)	0.728
mLOX	5/38	24,896/82,447	0.56 (0.21 – 1.48)	0.242
mLOY	5/53	22,735/95,293	0.95 (0.35 – 2.57)	0.922

[a] This indicates the number of subjects with at least one of loss, CN-LOH or gain in autosomal chromosomes.
[b] The p values were calculated with logistic regression analyses, using sex, age and age-squared as confounding factors. The p values in bold indicate statistically significant ($p < 0.05/6$ [= 8.33×10^{-3}]).

BBJ, BioBank Japan; CI, confidence interval; CN-LOH, copy-neutral loss of heterozygosity; mCAs, mosaic chromosomal alterations; mLOX, mosaic loss of the X chromosome; mLOY, mosaic loss of the Y chromosome; OR, odds ratio

Table S11. The results of logistic regression analyses between mCAs and postmortem interval

	OR (95% CI)	p value [a]
All autosomal mCAs [b]		
Suicide decedents [c]	1.13 (0.94 – 1.37)	0.203
Suicide decedents and non-suicide decedents [d]	1.12 (0.93 – 1.35)	0.228
Loss		
Suicide decedents [c]	1.20 (0.93 – 1.55)	0.160
Suicide decedents and non-suicide decedents [d]	1.16 (0.90 – 1.50)	0.257
CN-LOH		
Suicide decedents [c]	1.05 (0.81 – 1.37)	0.700
Suicide decedents and non-suicide decedents [d]	1.08 (0.84 – 1.38)	0.551
Gain		
Suicide decedents [c]	1.25 (0.85 – 1.84)	0.258
Suicide decedents and non-suicide decedents [d]	1.22 (0.83 – 1.81)	0.317
mLOX		
Suicide decedents [c]	1.05 (0.79 – 1.39)	0.730
Suicide decedents and non-suicide decedents [d]	1.06 (0.82 – 1.38)	0.653
mLOY		
Suicide decedents [c]	1.20 (0.96 – 1.49)	0.114
Suicide decedents and non-suicide decedents [d]	1.16 (0.93 – 1.45)	0.191

[a] The p values were calculated with logistic regression analyses, using sex, age and age-squared as confounding factors.
[b] This indicates the number of subjects with at least one of loss, CN-LOH or gain in autosomal chromosomes.
[c] In these, the distribution of PMI is as follows: 55 suicide decedents were within 6 hours, 145 were between 6 and 12 hours, 332 were between 12 and 24 hours, 154 were 24 and 48 hours, 24 were between 48 and 72 hours, and 28 were over 72 hours.
[d] In these, the distribution of PMI is as follows: 56 decedents were within 6 hours, 158 were between 6 and 12 hours, 357 were between 12 and 24 hours, 184 were 24 and 48 hours, 39 were between 48 and 72 hours, and 33 were over 72 hours.

CI, confidence interval; CN-LOH, copy-neutral loss of heterozygosity; mCAs, mosaic chromosomal alterations; mLOX, mosaic loss of the X chromosome; mLOY, mosaic loss of the Y chromosome; OR, odds ratio; PMI, postmortem interval

Table S12. Distribution of mCA events in suicide decedents and controls

	1st set		2nd set	
mCA events [a]	Suicide decedents (M/F)	Controls	Suicide decedents	Controls
0	311 (230/81)	80,139 (42,219/37,920)	240 (183/57)	80,056 (42,217/37,839)
1	88 (42/46)	7,721 (4,734/2,987)	95 (40/55)	7,761 (4,704/3,057)
2	10 (3/7)	834 (567/267)	22 (12/10)	864 (592/272)
3	1 (0/1)	112 (79/33)	2 (1/1)	143 (104/39)
4	0 (0/0)	34 (26/8)	1 (1/0)	20 (11/9)
5	0 (0/0)	13 (10/3)	1 (1/0)	13 (10/3)
6	0 (0/0)	7 (4/3)	0 (0/0)	6 (4/2)
7	0 (0/0)	5 (5/0)	1 (1/0)	3 (1/2)
8	0 (0/0)	0 (0/0)	0 (0/0)	0 (0/0)
9	0 (0/0)	2 (2/0)	0 (0/0)	2 (1/1)
10	0 (0/0)	2 (1/1)	0 (0/0)	1 (1/0)
11	0 (0/0)	0 (0/0)	0 (0/0)	0 (0/0)
12	0 (0/0)	0 (0/0)	0 (0/0)	0 (0/0)
13	0 (0/0)	0 (0/0)	1 (1/0)	1 (1/0)
14	0 (0/0)	0 (0/0)	0 (0/0)	0 (0/0)
15	0 (0/0)	0 (0/0)	0 (0/0)	0 (0/0)
16	0 (0/0)	1 (0/1)	0 (0/0)	0 (0/0)

[a] These indicate the numbers of mCA event in 1–22, X and Y chromosomes.
F, Females; M, Males; mCA, mosaic chromosomal alteration

Table S13. The results of logistic regression analyses and meta-analysis for mCAs in suicide death using only subjects with fewer than ten mCA events

	Number of subjects with mCA events/ number of subjects		OR (95% CI)	p value	P_{het} [a]
All autosomal mCAs [b]	Suicide decedents (mCA events < 10)	Controls			
1st set	37/410	8,728/88,867	1.42 (1.00 – 2.01)	0.0500 [c]	
2nd set	51/362	8,812/88,868	1.98 (1.45 – 2.71)	**$1.57{\times}10^{-5}$** [c]	
Combined			1.71 (1.36 – 2.16)	**$5.94{\times}10^{-6}$** [d]	0.159
Loss	Suicide decedents	Controls			
1st set	13/410	2,633/88,867	1.72 (0.98 – 3.01)	0.0600 [c]	
2nd set	26/362	2,665/88,868	3.27 (2.16 – 4.94)	**$1.85{\times}10^{-8}$** [c]	
Combined			2.61 (1.87 – 3.64)	**$1.62{\times}10^{-8}$** [d]	0.0697
CN-LOH	Suicide decedents	Controls			
1st set	20/410	5,267/88,867	1.18 (0.75 – 1.87)	0.467 [c]	
2nd set	21/362	5,235/88,868	1.22 (0.78 – 1.91)	0.386 [c]	
Combined			1.20 (0.87 – 1.66)	0.259 [d]	0.929
Gain	Suicide decedents	Controls			
1st set	6/410	1,416/88,867	1.57 (0.69 – 3.55)	0.280 [c]	
2nd set	11/362	1,483/88,868	2.48 (1.34 – 4.58)	**$3.84{\times}10^{-3}$** [c]	
Combined			2.10 (1.28 – 3.43)	**$3.10{\times}10^{-3}$** [d]	0.382
mLOX	Suicide decedents	Controls			
1st set	46/135	12,367/41,221	2.79 (1.85 – 4.22)	**$1.09{\times}10^{-6}$** [c]	
2nd set	64/123	12,529/41,224	4.39 (2.95 – 6.53)	**$3.04{\times}10^{-13}$** [c]	
Combined			3.53 (2.65 – 4.70)	**$5.83{\times}10^{-18}$** [d]	0.122
mLOY	Suicide decedents	Controls			
1st set	26/275	11,290/47,646	0.68 (0.44 – 1.04)	$7.79{\times}10^{-2}$ [c]	
2nd set	29/239	11,445/47,644	0.68 (0.44 – 1.04)	$7.60{\times}10^{-2}$ [c]	
Combined			0.68 (0.5 – 0.92)	$1.24{\times}10^{-2}$ [d]	0.992

[a] P for heterogeneity between two analyses (first set and second set) were calculated by Cochran's Q test.
[b] This indicates the number of subjects with at least one of loss, CN-LOH or gain in autosomal chromosomes.
[c] The p values were calculated with logistic regression analyses, using sex, age and age-squared as confounding factors.
[d] The p values were calculated with meta-analyses with the fixed-effect model. The p values in bold indicate statistically significant ($p < 0.05/6$ [$= 8.33 \times 10^{-3}$]).

CI, confidence interval; CN-LOH, copy-neutral loss of heterozygosity; mCAs, mosaic chromosomal alterations; mLOX, mosaic loss of the X chromosome; mLOY, mosaic loss of the Y chromosome; OR, odds ratio

Fig. S1. The results of principal component analysis (PCA)

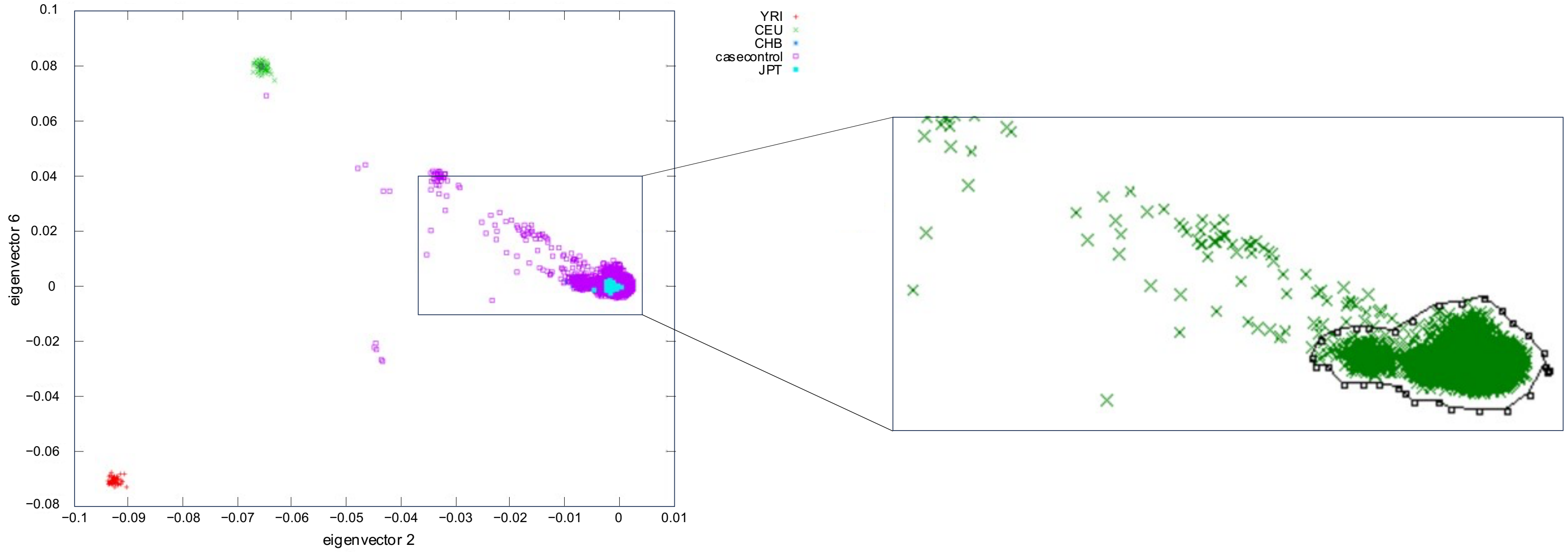

We mapped the BioBank Japan (BBJ) samples with 1KG populations and removed the samples that were PCA outliers from East Asian clusters in a plot.

Fig. S2. An overview of the study cohort

Main cohort

1st set

Suicide decedents (Kobe)
N = 410 (male 275, female 135)

Controls (BBJ)
N = 88,870 (male 47,647, female 41,223)

2nd set

Suicide decedents (Kobe)
N = 363 (male 240, female 123)

Controls (BBJ)
N = 88,870 (male 47,646, female 41,224)

Supplementary cohort

Healthy participants (Kobe)
N = 138 (male 77, female 61)

Non-suicide decedents (Kobe)
N = 91 (male 53, female 38)

Fig. S3. Distribution of detectable mosaicisms stratified by each autosomal chromosome

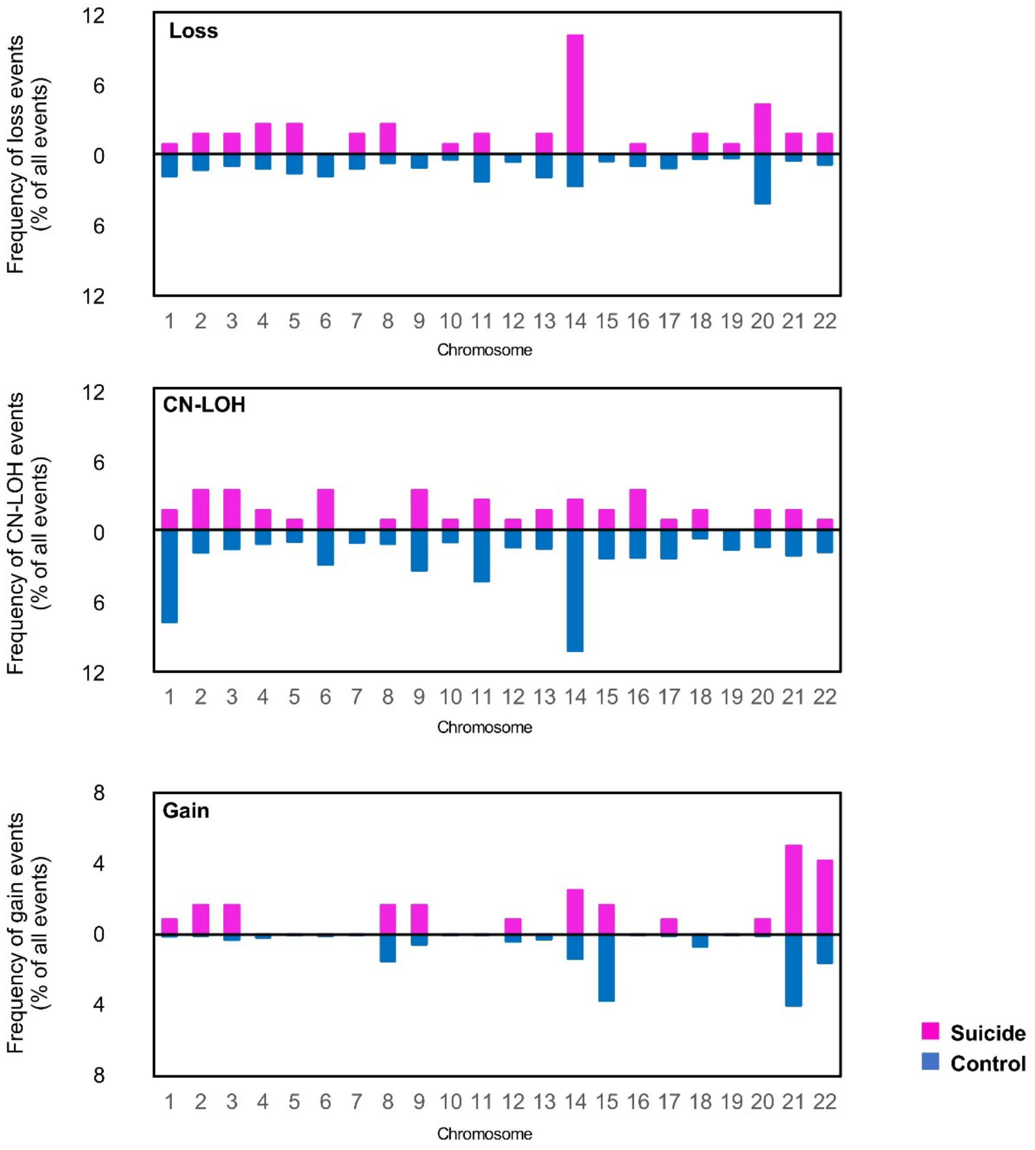

The vertical axis shows the frequency of occurrence of each chromosome, each mosaic out of the total autosomal mosaic occurrence in suicide decedents (magenta), and the control group (blue) of the integrated first and second sets. The statistical values of Spearman's correlation analysis between the two groups for the frequency of occurrence of each mCA (loss, CN-LOH, and gain) for each chromosome were as follows: R = 0.630, p = 1.45×10^{-8}.

CN-LOH, copy-neutral loss of heterozygosity; chr, chromosome; mCA, mosaic chromosomal alteration.

Fig. S4. Frequency of detectable mosaicism stratified by age, after excluding suicide decedents with diagnosed/possible psychiatric disorders

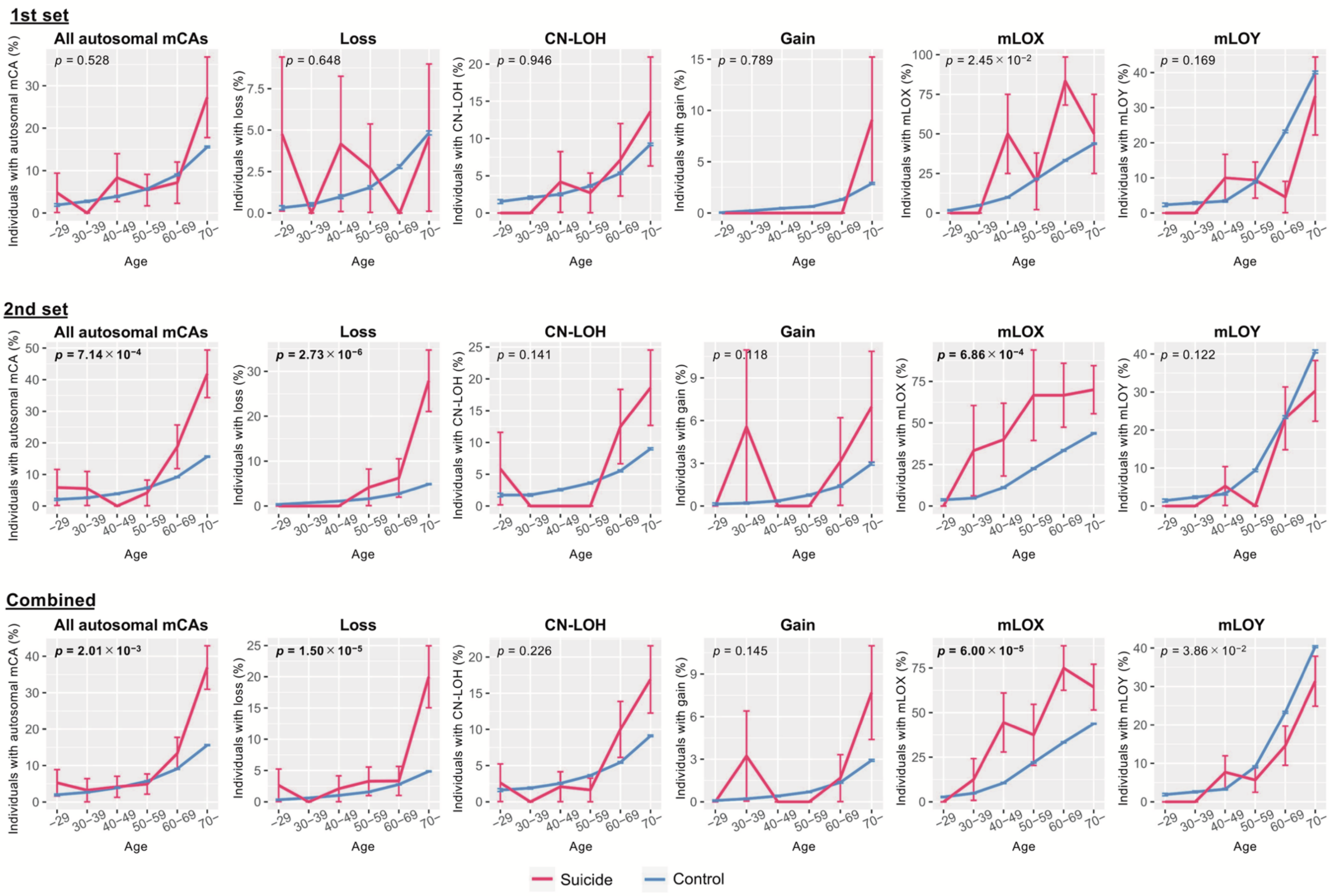

x-axis and y-axis represents age-category and the percentage of occurrence of each detectable mosaicism, respectively. Error bars indicate ± standard error. Blue lines denote control group, and magenta lines denote suicide decedents group. The p values for the 1st and 2nd set were calculated with logistic regression analyses adjusted with sex (for only autosomal mosaicisms), age, age-squared and array. The p values for the meta-analysis were calculated with fixed-effect model.

CN-LOH, copy-neutral loss of heterozygosity; mCA, mosaic chromosomal alteration; mLOX, mosaic loss of the X chromosome; mLOY, mosaic loss of the Y chromosome.

Fig. S5. Scatter plot of MR analysis inferring causal effect of mLOX and mLOY on suicide death

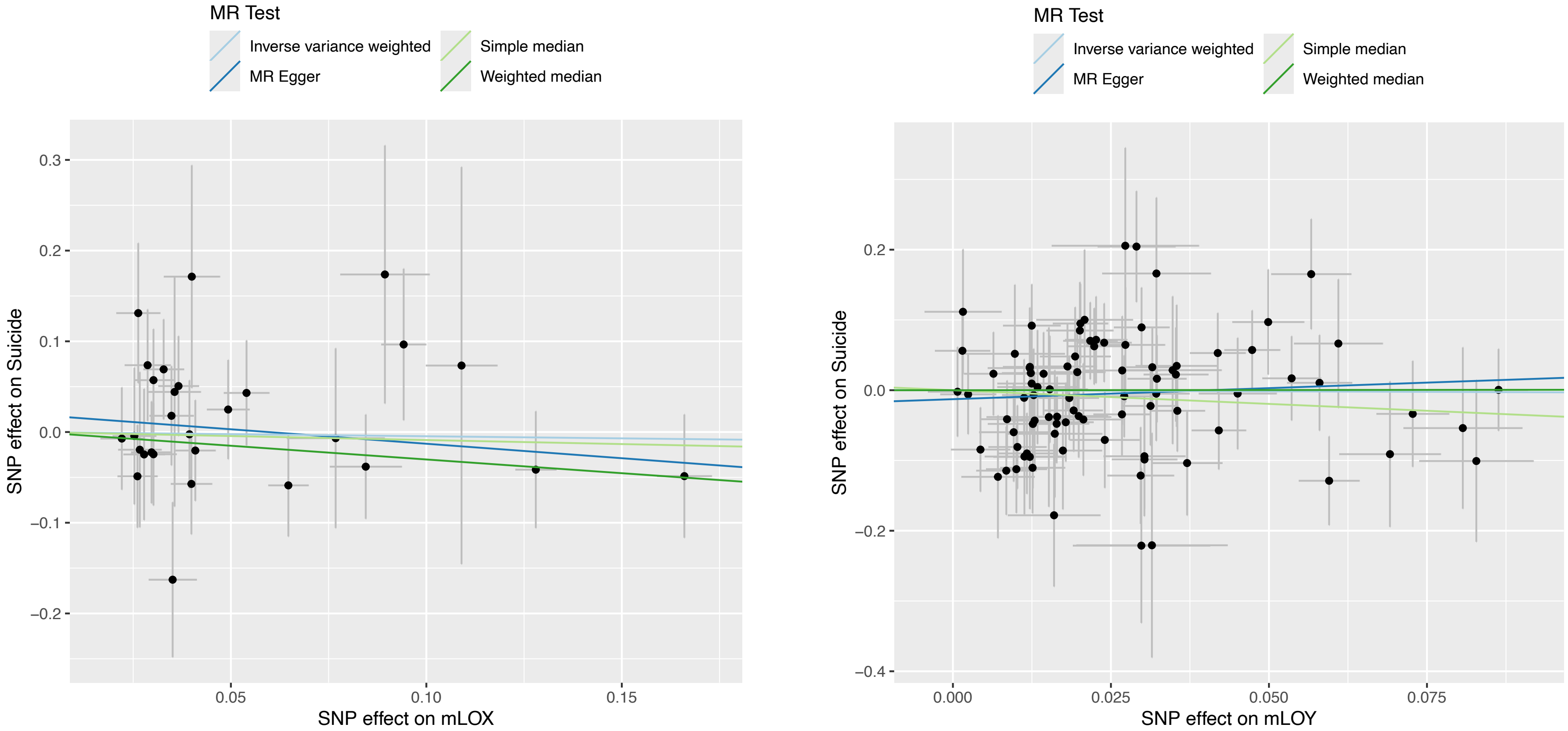

Scatter plots for the Mendelian randomization (MR) of the causal effect of mLOX or mLOY (exposure variable) on suicide death (outcome variable). Each dot represents the mLOX/mLOY-associated single nucleotide polymorphism (SNP) plotted along with the effect size estimates on mLOX/mLOY (x-axis) and suicide (y-axis). Error bars represent 95% confidence intervals. Coloured lines represent the causal association evaluated by four MR methods: inverse variance weighted (IVW); MR- Egger; simple median; weighted median. Full results can be seen in **Table S2**.
